# L-Dopa-modified microtubules lead to synapse instability in cultured neurons: possible implications in Parkinson’s disease therapy

**DOI:** 10.1038/s41531-025-01143-4

**Published:** 2025-10-17

**Authors:** Agustina Zorgniotti, Aditi Sharma, Sacnicte Ramirez-Rios, Chadni Sanyal, Martina Aleman, Yanina Ditamo, Marie-Jo Moutin, C. Gastón Bisig, Leticia Peris

**Affiliations:** 1 Univ. Grenoble Alpes, Inserm U1216, CNRS, Grenoble Institut des Neurosciences, Grenoble, France; 2 Centro de Investigaciones en Química Biológica de Córdoba (CIQUIBIC) - CONICET, Departamento de Química Biológica Ranwel Caputto, Facultad de Ciencias Químicas, Universidad Nacional de Córdoba, Córdoba, Argentina; 3 Department of Cell Developmental Biology, University of Pennsylvania Perelman School of Medicine, Philadelphia, PA USA

**Keywords:** Cellular neuroscience, Cell biology

## Abstract

L-Dopa, the main Parkinson’s disease treatment, unexpectedly alters the postsynaptic architecture of cultured mouse neurons through its incorporation into α-tubulin via tubulin tyrosine ligase (TTL). This leads to the formation of L-Dopa-modified microtubules, with impaired dynamics and reduced entry into dendritic spines, increasing their vulnerability to pruning. As a result, dendritic spine density—particularly mature spines—and the number of excitatory synapses are significantly reduced. L-Dopa-mediated synaptic defects are absent in neurons lacking the ability to incorporate it into α-tubulin—TTL or SVBP knockout neurons—confirming a microtubule-dependent mechanism. In vitro, L-Dopa-modified tubulin interfered with VASH1-SVBP activity, the major brain’s tubulin carboxypeptidase, potentially prolonging L-Dopa-microtubule persistence and turning this therapeutic agent into a driver of long-lasting neuronal changes. These findings reveal a novel mechanism of L-Dopa-induced synaptotoxicity—mediated by the disruption of microtubule dynamics and the tubulin tyrosination/detyrosination cycle—with possible implications for the long-term adverse effects of L-Dopa therapy.

## Introduction

Parkinson’s disease is a neurodegenerative disorder characterized by progressive degeneration of the dopaminergic nigrostriatal pathway, leading to slowing of movement, rigidity and tremor, as well as cognitive problems and increased risk of dementia and depression^1,2^. To date, the administration of L-3,4-dihydroxyphenylalanine (L-Dopa), the immediate precursor of dopamine, is the gold-standard pharmacological therapy for Parkinson’s disease. L-Dopa mitigates motor symptoms, but its charm comes with a dark twist: over time, its beneficial effect is tarnished by the appearance of motor fluctuations and abnormal and dyskinetic movements, known as L-Dopa-induced dyskinesia. The longer the treatment with L-Dopa, the more it gives way to fluctuations in motor response and involuntary movements, together with affective and cognitive disturbances^3^. Despite extensive exploration of the underlying mechanics of these motor and psychiatric complications^3,4^, the true nature of what triggers this phenomenon at the molecular level remains elusive.

In Parkinson’s disease patients, as well as in various experimental models, it has been observed that prolonged treatment with L-Dopa disrupts the delicate balance of synaptic mechanisms, triggering maladaptive changes in both the function and structure of synapses, particularly in striatal medium spiny neurons and corticostriatal connections^5,6^. The emerging consensus is that, as the disease progresses, alteration in dopamine release and reuptake, and abnormalities in neurotransmitter systems, are involved in the development of L-Dopa-induced dyskinesia^7^. Intriguingly, even in primate models without nigrostriatal damage, chronic administration of high doses of L-Dopa can induce dyskinesia, highlighting the involvement of L-Dopa per se in these effects and the complex interplay between treatment and pathology^8^.

Over the past decade, research has demonstrated that synaptic function and structure rely on the neuronal cytoskeleton, including actin filaments and microtubules. Microtubule dynamics, defined by their stochastic transitions between depolymerization and polymerization phases—a process known as dynamic instability^9^—critically contributes to synaptic structure and function within both pre- and postsynaptic compartments^10^. Dendritic spines are excitatory post-synaptic structures where glutamatergic and dopaminergic signals are integrated. While the dendritic spine structure is mostly supported by actin filaments, dynamic microtubules originating from the dendritic shaft sporadically invade the spines^11,12^. Microtubule invasion directly influences the regulation of spine composition and morphology, playing an important role in synapse function and plasticity^12–14^. Microtubule entry into spines is dependent on synaptic activity, Ca^2+^ influx, actin polymerization, and post-translational modifications of tubulin, and correlates with long-lasting morphological changes of spines and synaptic strength^15,16^.

Microtubule dynamics are modulated by post-translational modifications such as the cyclic detyrosination/tyrosination of α-tubulin^17–19^. Upon microtubule assembly, the tyrosine residue encoded at the C-terminal α-tubulin (Tyr-tubulin) is removed by the carboxypeptidase enzymes—heterodimeric complexes of small vasohibin binding protein (SVBP) with vasohibin 1 (VASH1) or vasohibin 2 (VASH2)^20,21^, and the recently described microtubule-associated tyrosine carboxypeptidase (MATCAP)^22^—to generate detyrosinated tubulin (deTyr-tubulin)^23^. Following microtubule depolymerization, tubulin tyrosine ligase (TTL) rapidly tyrosinates soluble deTyr-tubulin, which can reassemble into tyrosinated newly formed microtubules^24^. The pool of deTyr-tubulin can be further processed by cytosolic carboxypeptidases that remove the terminal glutamate. This generates Δ2 tubulin, which, unlike deTyr-tubulin, cannot be re-tyrosinated by TTL^25,26^, but can be further processed by cytosolic carboxypeptidases 1, 4, 5 and 6, to generate Δ3 tubulin. Tyr-tubulin is associated with dynamic microtubules^24,26^; in contrast, deTyr- and Δ2-tubulin are associated with more stable, longer-lived microtubules^27–29^. Δ3 tubulin is present in dynamic microtubules and represents a small proportion (around 1%) of the C-terminal variants of α-tubulin in the mouse brain^30^. The role of tubulin detyrosination/tyrosination in many physiological processes remains poorly understood; however, there is increasing evidence of its importance in various specialized microtubule functions^16,31–34^ as the presence or absence of a C-terminal tyrosine residue on α-tubulin affects microtubule interaction with molecular motors^29,35–37^ and microtubule-associated proteins^38^.

A balanced tubulin detyrosination/tyrosination cycle is essential for neurons and brain integrity, as demonstrated by lethal brain and neuronal defects in TTL knockout mice^39–42^. Furthermore, TTL heterozygous mice exhibit a reduction in dendritic spine density, impaired synaptic plasticity, and memory deficits, which are attributed to the partial increase in tubulin detyrosination^16^. On the other hand, defective tubulin detyrosination due to the absence of VASH-SVBP carboxypeptidases causes structural brain abnormalities and cognitive deficiencies in both humans and mice^31,43,44^.

Disruption of the balance between tubulin detyrosination/tyrosination can also occur through the incorporation of tyrosine analogs like phenylalanine^45^, which is abundant in phenylketonuric patients, or L-Dopa^46^, widely used in Parkinson’s disease treatment. Our previous studies demonstrated that L-Dopa can be incorporated by TTL into the α-tubulin C-terminus, both in vitro and in living cells, leading to a non-physiological post-translational modification of microtubules that alters their dynamics, impairs mitochondrial transport, and reduces the ability of the molecular motor KIF5B to bind to L-Dopa-modified microtubules^46,47^. Remarkably, unlike tyrosine, once integrated into microtubules, L-Dopa resists its removal by tubulin carboxypeptidases under conditions that typically prompt rapid tyrosine release^47^. As a result, L-Dopa-tubulin accumulates over time, potentially transforming the therapeutic agent into a source of long-lasting abnormal microtubule modification.

Here, combining primary hippocampal neurons—wild type or lacking either of the detyrosination/tyrosination cycle enzymes—super-resolution live cell imaging and single-molecule total internal reflection fluorescence (TIRF) microscopy assays, we evaluate the synaptic consequences of L-Dopa-microtubules presence and describe the molecular basis of their abnormal interaction with the VASH1-SVBP complex. We show that L-Dopa incorporation into microtubules reduces dendritic spine density and excitatory synapses in wild-type hippocampal neurons, without altering spine morphology. Interestingly, this reduction was not observed in TTL KO neurons, where L-Dopa cannot be incorporated into α-tubulin due to the absence of the ligase TTL, nor in SVBP KO neurons, where reduced levels of detyrosinated α-tubulin—that acts as substrate for L-Dopa incorporation—impairs its addition, indicating that the defects observed in wild type neurons are microtubule-dependent. We show that the presence of L-Dopa-modified microtubules disrupts both the binding and the activity of VASH1-SVBP, the most abundant carboxypeptidase in the brain. We also demonstrate that this non-physiological post-translational modification of microtubules modifies their overall dynamics, thereby reducing microtubule invasion into dendritic spines and making the spines more susceptible to pruning.

Our results suggest that, concomitantly with the beneficial enhancement of dopamine synthesis, the tyrosination state of tubulin (and microtubules) could be gradually altered by the non-physiological incorporation of L-Dopa into α-tubulin C-terminus. Additionally, the inability of endogenous carboxypeptidases to remove L-Dopa from microtubules, combined with the chronic nature of the treatment, could exacerbate its synaptotoxic effects. This alteration may disrupt essential processes for maintaining synaptic connectivity and functionality, potentially contributing to neurological deficits observed after prolonged L-Dopa therapy, offering new insights into the changes caused by this treatment in patients with Parkinson’s disease.

## Results

### L-Dopa incorporation into microtubules reduces dendritic spine density and excitatory synapses in wild type hippocampal neurons

**Fig. 1:**
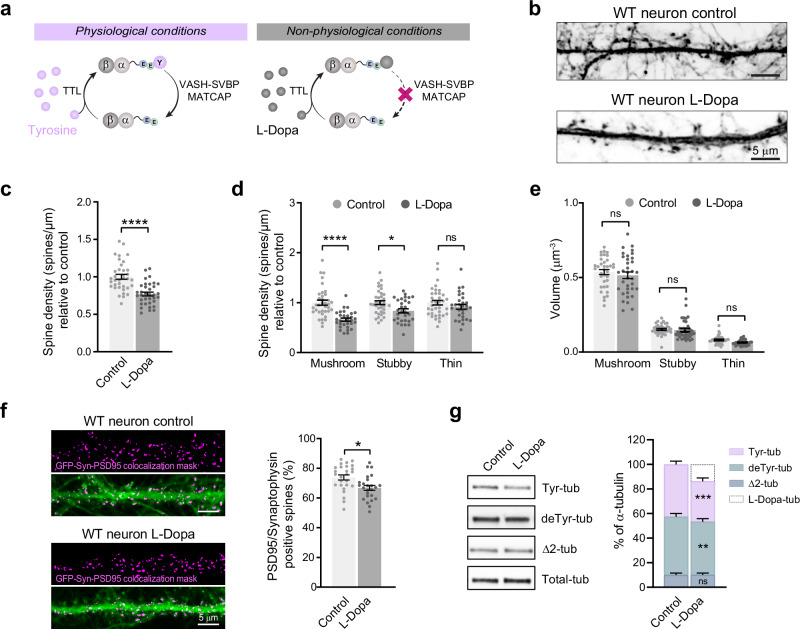
L-Dopa incorporation into microtubules reduces dendritic spine density and excitatory synapses in cultured hippocampal neurons. **a** Schematic representation of αβ-tubulin dimers with their C-terminal tails. Under physiological conditions (left), tubulin carboxypeptidases (VASH1/2-SVBP and MATCAP) remove the C-terminal tyrosine (Y, in violet) from α-tubulin. Tubulin tyrosine ligase (TTL) re-adds tyrosine at the C-terminal glutamate (E, in green) of detyrosinated tubulin. In the presence of L-Dopa (right, in gray), this amino acid is incorporated into detyrosinated tubulin by the TTL enzyme, replacing the tyrosine residue in the detyrosination/tyrosination cycle. **b** Confocal images of eGFP-expressing wild type hippocampal neurons (18 DIV) treated with L-Dopa (0.4 mM) or the vehicle (control). Scale bar: 5 µm. **c**, **d** Dendritic spine density. Values were normalized to the mean of the control cells. **c** Total spine density: Student’s *t*-test; ****p < 0.0001. **d** Spine density of each morphological type of spine. **e** Dendritic spine volume. Graphs represent mean ± SEM; *n* = 36 control and *n* = 35 L-Dopa-treated neurons from three independent experiments. Two-way ANOVA; ****p < 0.0001; *p < 0.05. **f** Confocal images of magnified dendritic segments from control and L-Dopa-treated hippocampal neurons expressing soluble GFP (green). Images are superposed with a mask (colocalization mask, magenta) corresponding to pixels that simultaneously represent PSD-95, synaptophysin and GFP labels. Scale bar: 5 µm. Percentage of dendritic spines containing fluorescent puncta. Data represent mean ± SEM; *n* = 26 control and *n* = 25 L-Dopa-treated neurons from three independent experiments. Kruskal–Wallis test; *p < 0.05. **g** Representatives immunoblot of protein extracts from wild type hippocampal neurons (17 DIV) treated with L-Dopa (0.4 mM, 1 h) or the vehicle (control) showing tyrosinated- (Tyr-tub), detyrosinated- (deTyr-tub), Δ2- (Δ2-tub) and total- (Total-tub) α-tubulin levels. The content of the different α-tubulin forms was estimated after normalization to total α-tubulin levels and antibody sensitivity, as described in the “Methods” section. Graphs represent mean ± SEM; *n* = 14 neuron samples from four different experiments. Paired *t*-test; ***p < 0.001; **p < 0.01.

Previous studies have demonstrated that L-Dopa, a tyrosine analog commonly used as standard treatment in Parkinson’s disease, can be incorporated by the TTL enzyme at the C-terminal tail of α-tubulin, instead of the tyrosine residue, which can further polymerize into microtubules^46,47^ (Fig. 1a). In the present study, we used hippocampal neurons as a model system based on the fact that these cells lack the ability to convert L-Dopa into dopamine, ensuring that the administered L-Dopa remains unmodified and available for incorporation into tubulin.

Mature wild type hippocampal neurons treated with L-Dopa (0.4 mM, 1 h) showed reduced dendritic spine density (Fig. 1b, c; 1.00 ± 0.03 spines/µm vs 0.77 ± 0.02 spines/µm for control and L-Dopa treated, respectively), affecting mainly the mature forms of dendritic spines (Fig. 1d; 34 ± 10% vs 16 ± 9% of reduction in mushrooms and stubby spines, respectively), indicating that L-Dopa incorporation into tubulin and formation of L-Dopa-microtubules had deleterious consequences on the maintenance of the dendritic spines. The volume of each type of spine was unaffected after L-Dopa treatment (Fig. 1e), suggesting that L-Dopa treatment does not perturb final spine morphology, but rather induces spine pruning and/or changes from one spine subpopulation to another.

To determine whether changes in dendritic spines were associated with synaptic alterations, we quantified excitatory synapses in cultured hippocampal neurons^48^. After 18 days in vitro (DIV), the cells, transduced with lentiviral vectors to express soluble GFP, were treated with L-Dopa, fixed and immunostained with anti-PSD-95 and anti-synaptophysin antibodies (postsynaptic and presynaptic marker, respectively, Supplementary Fig. 1). Fluorescent puncta containing both pre- and postsynaptic markers were used to detect and count synapses formed on the spines of transduced cells, as shown in Fig. 1f. Neurons treated with L-Dopa displayed a reduced percentage of spines containing excitatory synapses (73 ± 2% of spines vs 66 ± 2% of spines in control and L-Dopa treated neurons, respectively). Consequently, L-Dopa treatment not only decreases dendritic spine density but also the percentage of excitatory synapses in the remaining spines. These results suggest that L-Dopa treatment may induce a cumulative defect in the synaptic compartment that would amplify the gravity of the synaptic phenotype.

The analysis of the tyrosination state of tubulin on these neurons demonstrated that L-Dopa treatment decreases Tyr-tubulin levels by almost 10% and deTyr-tubulin levels by 5% (Fig. 1g, Supplementary Fig. 2; 43 ± 3% vs 33 ± 3% and 47 ± 3% vs 43 ± 2% of total α-tubulin, for Tyr- and deTyr-tubulin in control and L-Dopa treated neurons, respectively) with no significant change in the levels of Δ2-tubulin or Δ3-tubulin (Fig. 1g, Supplementary Figs. 2, 3). This indicates the presence of a new tubulin pool that is recognized by total α-tubulin antibody, but is neither tyrosinated-, detyrosinated-, nor Δ2-tubulin. Although a specific L-Dopa-tubulin antibody is not available, we have previously confirmed the presence of L-Dopa at the C-terminus of α-tubulin^46,47^. Therefore, we assume that this newly observed tubulin pool that appears after L-Dopa treatment consists of L-Dopa-tubulin and corresponds to approximately 15% of the total tubulin present in wild type neurons (Fig. 1g).

### VASH1-SVBP carboxypeptidase complex shows reduced activity and altered binding behavior as a consequence of L-Dopa incorporation into microtubules

**Fig. 2:**
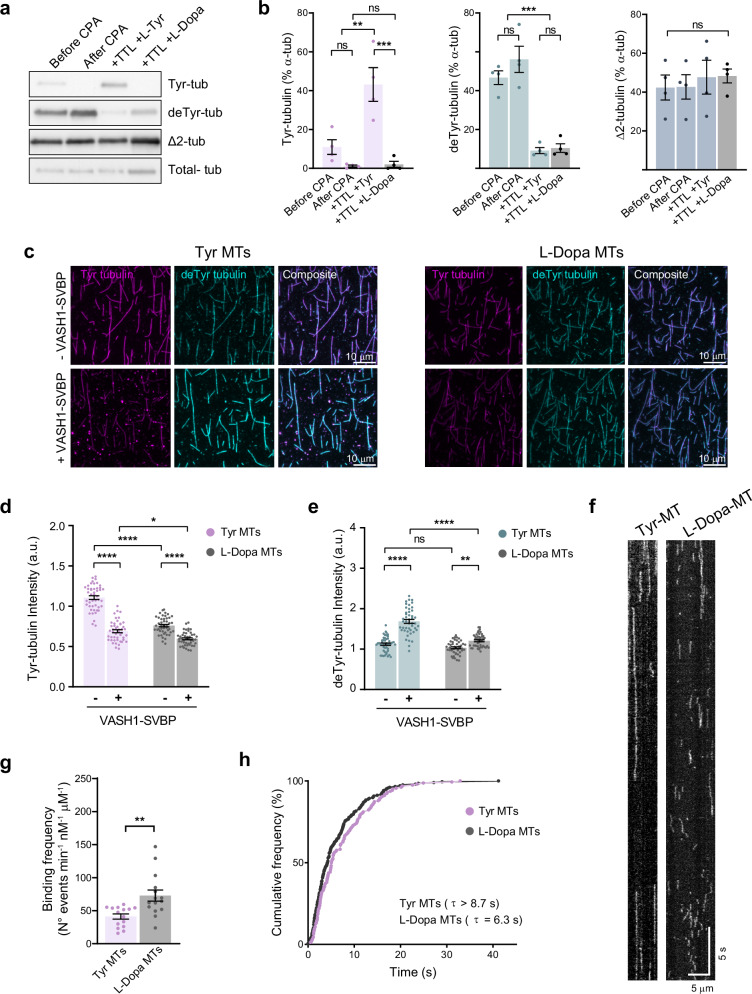
VASH1-SVBP shows reduced activity and altered binding behavior on L-Dopa-enriched microtubules. **a** Representative immunoblot of purified bovine brain tubulin showing tyrosinated- (Tyr-tub), detyrosinated- (deTyr-tub), Δ2- (Δ2-tub) and total- (Total-tub) α-tubulin levels, in the initial tubulin preparation (Before CPA), after the incubation with pancreatic carboxypeptidase A (After CPA) and following the incubation with TTL in the presence of L-Tyrosine (+ TTL + L-Tyr) or L-Dopa (+ TTL + L-Dopa). **b** The content of the different α-tubulin forms was estimated after normalization to the corresponding α-tubulin levels and expressed as the percentage of total α-tubulin in each fraction. The graphs show the mean ± SEM from four independent experiments. One-way ANOVA; **p < 0.01, ***p < 0.001. **c** Representative images of microtubules enriched in tyrosinated (Tyr MTs) or L-Dopa (L-Dopa MTs) tubulin. Tyrosinated (Tyr, magenta) and detyrosinated (deTyr, cyan) α-tubulin pools after 30 min of incubation in the absence (-VASH1-SVBP) or presence (+VASH1-SVBP) of 50 pM VASH1-SVBP complex are shown. Scale bar 10 µm. **d**, **e** Analysis of tyrosinated (**d**) and detyrosinated (**e**) tubulin signal intensities. Values were normalized to the mean of the tyrosinated microtubules in the absence of enzyme. Each point represents an individual microtubule in a typical experiment. Data represent mean ± SEM; *n* = 46 microtubules per condition. Kruskal–Wallis test; ****p < 0.0001, **p < 0.01, *p < 0.05. **f** Representative kymographs of single molecules of catalytically inactive sfGFP-tagged VASH1–SVBP (deadVASH1-SVBP, 25 pM) bound to Taxol-stabilized microtubules enriched in tyrosinated (Tyr MTs) or L-Dopa (L-Dopa MTs) tubulin. Scale bars: horizontal, 5 µm; vertical, 5 s. **g** Binding frequency of deadVASH1-SVBP enzyme to tyrosinated (Tyr MTs) or L-Dopa (L-Dopa MTs) microtubules. **h** Cumulative frequency of the residence times measured in TIRF movies taken during the 30 min following addition of enzyme complexes to tyrosinated (Tyr MTs) or L-Dopa (L-Dopa MTs) microtubules. The mean residence time (τ) is obtained by fitting the curve with a mono-exponential function, as previously described^49^. Each point represents an individual microtubule in a typical experiment. Data represent mean ± SEM; *n* = 15 microtubules per condition. Student’s *t*-test; **p < 0.01.

We previously demonstrated with in vitro studies that L-Dopa incorporated at the α-tubulin C-terminus cannot be released by endogenous tubulin carboxypeptidase from rat brain extract, which subsequently inhibits the incorporation of tyrosine^47^. We now analyzed the release of L-Dopa from microtubules by evaluating the activity of recombinant VASH1-SVBP complex—the principal detyrosinase enzyme in the brain—on L-Dopa-enriched microtubules by in vitro biomimetic assays. To generate Tyr- or L-Dopa-modified microtubules, we first induced α-tubulin detyrosination by incubating purified bovine brain tubulin with pancreatic carboxypeptidase A (CPA). After CPA inactivation, the obtained deTyr-tubulin was incubated with recombinant TTL enzyme and either L-Tyrosine or L-Dopa (Fig. 2a). As expected, we observed a significant increase in Tyr-tubulin levels when deTyr-tubulin was incubated with TTL and L-Tyrosine, and no changes when incubated with TTL and L-Dopa (Fig. 2b, from 1.2 ± 0.5% to 43 ± 9% vs 2 ± 1% for TTL +Tyr and TTL + L-Dopa, respectively). We observed an important reduction of deTyr-tubulin levels in both conditions (from 56 ± 7% to 9 ± 1% vs 10 ± 2% for TTL +Tyr and TTL + L-Dopa, respectively) with no major changes in the levels of Δ2-tubulin (from 43 ± 6% to 48 ± 9% vs 48 ± 3% for TTL +Tyr and TTL + L-Dopa, respectively, Fig. 2b). The reduction of deTyr-tubulin levels with no change in Tyr- and Δ2-tubulin in TTL + L-Dopa condition provided first-time evidence of L-Dopa incorporation into tubulin by TTL in a cell-free system reconstituted from purified proteins.

We next analyzed the detyrosinating activity of the recombinant VASH1-SVBP complex in vitro. We immobilized Taxol-stabilized microtubules, enriched in tyrosinated or L-Dopa tubulin (Tyr-MTs or L-Dopa-MTs), on the surface of TIRF chambers, as previously described^49^, and tested the activity of the complex by immunofluorescence (Fig. 2c, Table 1). In control conditions, without purified VASH1-SVBP, the Tyr-tubulin content in Tyr-MTs was significantly higher than in L-Dopa-MTs (1.11 ± 0.02 a.u. vs 0.76 ± 0.02 a.u. for Tyr- and L-Dopa-MTs, respectively, Fig. 2d) while the deTyr-tubulin content of both was similar (1.12 ± 0.03 a.u. and 1.03 ± 0.02 a.u. for Tyr- and L-Dopa-MTs, respectively; Fig. 2e). After incubation with VASH1-SVBP, as expected, the reduction in Tyr-tubulin content in Tyr-MTs was greater than in L-Dopa-MTs (38 ± 5% vs 21 ± 5%, respectively; Fig. 2d). Interestingly, VASH1-SVBP increased the deTyr-tubulin content 3-times more in Tyr-MTs than in L-Dopa-MTs (51 ± 6% and 17 ± 4%, respectively; Fig. 2e), clearly indicating a reduced carboxypeptidase activity of VASH1-SVBP on the L-Dopa enriched microtubules compared to Tyr-MTs.

**Table 1:** Analysis of tyrosinated and detyrosinated tubulin levels on Tyr- and L-Dopa-enriched microtubules

Experiment	Microtubule	VASH1-SVBP (50 pM)	Tyr-tub intensity (a.u.)	deTyr-tub intensity (a.u.)
**a**	Tyr-MTs	-	1.11 ± 0.02	1.12 ± 0.03
		+	0.69 ± 0.02	1.69 ± 0.05
	L-Dopa-MTs	-	0.76 ± 0.02	1.03 ± 0.02
		+	0.60 ± 0.01	1.21 ± 0.02
**b**	Tyr-MTs	-	0.90 ± 0.02	1.00 ± 0.03
		+	0.85 ± 0.02	1.64 ± 0.02
	L-Dopa-MTs	-	0.70 ± 0.01	0.77 ± 0.01
		+	0.61 ± 0.01	0.87 ± 0.01
**c**	Tyr-MTs	-	1.00 ± 0.02	1.00 ± 0.03
		+	0.25 ± 0.01	1.94 ± 0.05
	L-Dopa-MTs	-	0.67 ± 0.02	1.48 ± 0.03
		+	0.27 ± 0.01	2.12 ± 0.05

Tyrosinated and detyrosinated tubulin signal intensities corresponding to tyrosinated- or L-Dopa-enriched microtubules after 30 min of incubation in the absence (-) or presence (+) of the VASH1-SVBP complex. Results correspond to experiments performed on different days, using different protein preparations. The values were normalized to the mean of the tyrosinated microtubules in the absence of enzyme. Data represent mean ± SEM; at least 40 microtubules per condition were analyzed. (**a**) Related to Fig. 2.

In order to understand the molecular mechanisms involved in the distinct detyrosination activity of VASH1-SVBP on L-Dopa-microtubules, we examined the microtubule-interacting behavior by single-molecule TIRF microscopy. Microtubule detyrosination has been shown to impact the interaction between VASH-SVBP complexes and microtubules^49^; therefore, to enable a detailed analysis of enzyme-microtubule interactions without ongoing microtubule modification throughout the experiment, we opted to use a catalytically inactive version of the VASH1–SVBP complex (deadVASH1-SVBP^49^). We first tested the same enzyme concentration used for activity experiments. Interestingly, as shown in representative kymographs, the enzyme complex exhibited significantly different binding behavior on the two types of microtubules (Table 2, Supplementary Fig. 4); however, to minimize bias in quantification caused by the high number of bound molecules, we reduced the enzyme concentration to 25 pM (Fig. 2f, Table 2). We observed that deadVASH1-SVBP bound more frequently on L-Dopa-MTs than on Tyr-MTs (73 ± 8 vs 41 ± 4 events.min^-1^.µm^-1^.nM^-1^, respectively; Fig. 2g, Table 2) with shorter residence times on L-Dopa-MTs (τ = 6.3 s vs τ > 8.7 s for L-Dopa- and Tyr-MTs, respectively; Fig. 2h, Table 2). It should be noted that, as many molecules were already attached to the microtubules at the start of the films, the residence time of the enzyme on Tyr microtubules could not be accurately determined and is underestimated (Fig. 2f).

**Table 2:** Binding parameters of VASH1-SVBP complex interaction with tyrosinated- and L-Dopa-enriched microtubules

Experiment	Recombinant protein (concentration)	Microtubule	Residence time τ (s)	Binding frequency (min^-1^.nM^-1^.µm^-1^)
**a**	deadVASH1-SVBP (50 pM)	Tyr-MTs	2.2	72 ± 5
		L-Dopa-MTs	1.5	130 ± 7
**b**	deadVASH1-SVBP (25 pM)	Tyr-MTs	8.7	65 ± 7
		L-Dopa-MTs	6.3	85 ± 9
**c**	deadVASH1-SVBP (25 pM)	Tyr-MTs	9.5	41 ± 4
		L-Dopa-MTs	3.6	73 ± 8

Resident time and binding frequency results corresponding to experiments performed on different days, using different protein preparations. At least 15 microtubules per condition were analyzed. (**a**) Related to Supplementary Fig. 2. (**b**, **c**) Related to Fig. 2.

Taken together, these results highlight the differential binding behavior of the VASH1-SVBP enzyme on Tyr- and L-Dopa-microtubules that might contribute to the reduction in its carboxypeptidase activity. These abnormalities could contribute to the prolonged persistence of L-Dopa-modified-microtubules in cells, exacerbating their atypical behavior and leading to a cumulative impact over time.

### The synaptic defects induced by L-Dopa treatment are dependent on its incorporation into microtubules

**Fig. 3:**
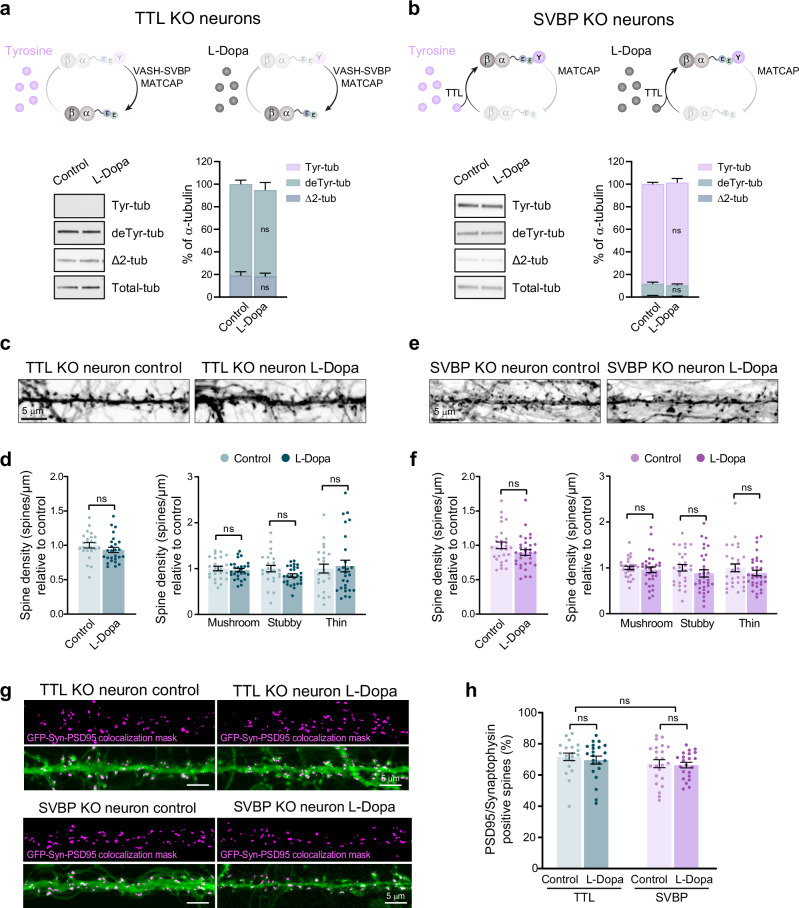
L-Dopa treatment does not modify dendritic spine density in TTL KO or SVBP KO hippocampal neurons. **a**, **b** Schematic representation of the detyrosination/tyrosination cycle in the presence of tyrosine (violet) or L-Dopa (gray) and representative immunoblot of protein extracts from TTL KO (**a**) and SVBP KO (**b**) hippocampal neurons (17 DIV) treated with L-Dopa (0.4 mM, 1 h) or the vehicle (control), showing tyrosinated- (Tyr-tub), detyrosinated- (deTyr-tub), Δ2- (Δ2-tub) and total- (Total-tub) α-tubulin levels. The content of the different α-tubulin forms was estimated after normalization to total α-tubulin levels and antibody sensitivity, as described in the “Methods” section. Paired *t*-test; *n* = 9 TTL KO neuron samples from three independent experiments and *n* = 4 SVBP KO neuron samples from two independent experiments. **c**–**f** Confocal images of eGFP-expressing TTL KO (**c**) and SVBP KO (**e**) hippocampal neurons (18 DIV) treated with 0.4 mM L-Dopa or the vehicle (control). Scale bar: 5 µm. Total dendritic spine density, or that of each different morphological type of spine, is represented for TTL KO (**d**) and SVBP KO (**f**) neurons. Spine density values were normalized to the mean of the control cells. Graphs represent mean ± SEM; *n* = 24 control and *n* = 27 L-Dopa-treated TTL KO neurons and *n* = 29 control and *n* = 32 L-Dopa-treated SVBP KO neurons from three independent experiments. Student’s *t*-test and two-way ANOVA for total and morphologically classified density, respectively. **g** Representative confocal images of control and L-Dopa-treated hippocampal TTL KO and SVBP KO neurons (DIV18) expressing soluble GFP (green). Signal overlap mask (colocalization mask, magenta) corresponds to pixels that simultaneously represent PSD-95 (post-synaptic marker), synaptophysin (pre-synaptic marker) and GFP label. Scale bar: 5 µm. **h** Percentage of dendritic spines containing fluorescent puncta of both pre- and post-synaptic markers in GFP-expressing neurons. Data represent mean ± SEM; *n* = 20, *n* = 22 control and L-Dopa-treated TTL KO neurons; and *n* = 23, *n* = 22 control and L-Dopa-treated SVBP KO neurons, respectively; from at least two independent experiments. Kruskal–Wallis test.

To confirm that dendritic spine defects observed in wild type neurons after L-Dopa treatment were microtubule-dependent, we used two cellular models in which there is no post-translational incorporation of L-Dopa into tubulin due to the absence of either of the key enzymes of the detyrosination/tyrosination cycle: TTL KO and SVBP KO hippocampal cultured neurons (Fig. 3a, b).

In TTL KO neurons, neither tyrosine nor L-Dopa can be incorporated into the C-terminus of α-tubulin due to the absence of the ligase enzyme (Fig. 3a). In these neurons, deTyr- and Δ2-tubulin levels were unaffected by the treatment with L-Dopa (81 ± 4% vs 77 ± 7% for deTyr-tubulin; 19 ± 4% vs 18 ± 3% for Δ2-tubulin in control and L-Dopa treated neurons, respectively; Fig. 3a, Supplementary Fig. 2). As expected, L-Dopa treatment did not affect dendritic spine density (1.00 ± 0.04 spines/µm vs 0.93 ± 0.04 spines/µm for control and L-Dopa treated neurons, respectively; Fig. 3c, d) or dendritic spine subpopulation (1.00 ± 0.05 spines/µm vs 0.97 ± 0.04 spines/µm, 1.00 ± 0.07 spines/µm vs 0.85 ± 0.04 spines/µm, 1.0 ± 0.1 spines/µm vs 1.0 ± 0.1 spines/µm for mushroom, stubby and thin spines in control and L-Dopa treated neurons; Fig. 3d), indicating that the post-translational incorporation of L-Dopa into tubulin through TTL was necessary for the reduction in dendritic spine density observed in wild type neurons.

As L-Dopa needs deTyr-tubulin to be incorporated into microtubules, we also used cultured hippocampal neurons from the SVBP KO model. It has been shown that the absence of the chaperone SVBP results in a complete absence of detyrosinase activity for VASH1/2 enzymes, and a drastic reduction of deTyr-tubulin levels in cells^20,21,31^ (Fig. 3b). As expected, L-Dopa treatment did not significantly modify the levels of Tyr-, deTyr- or Δ2-tubulin in SVBP KO cultured neurons (88 ± 2% vs 91 ± 4%; 10 ± 2% vs 9 ± 1% and 1.0 ± 0.2% vs 0.9 ± 0.1% for Tyr- deTyr- and Δ2-tubulin in control and L-Dopa treated neurons, respectively; Fig. 3b, Supplementary Fig. 2), most likely due to a reduction in the substrate for the TTL enzyme, which limits the incorporation of L-Dopa into tubulin. Consequently, L-Dopa treatment did not affect dendritic spine density nor dendritic spine subpopulation in SVBP KO cultured hippocampal neurons (1.00 ± 0.05 spines/µm vs 0.89 ± 0.04 spines/µm for control and L-Dopa treated neurons, respectively; 1.00 ± 0.04 spines/µm vs 0.96 ± 0.06 spines/µm, 1.00 ± 0.07 spines/µm vs 0.88 ± 0.08 spines/µm, and 1.00 ± 0.08 spines/µm vs 0.89 ± 0.06 spines/µm for mushroom, stubby and thin spines in control and L-Dopa treated neurons, respectively; Fig. 3e, f).

As in wild type neurons L-Dopa treatment not only induced a reduction in dendritic spine density but also a decrease in the proportion of excitatory synapses, we analyzed by immunofluorescence the percentage of spines containing both the pre- and postsynaptic markers (synaptophysin and PSD95, respectively), in GFP expressing TTL KO and SVBP KO neurons (Fig. 3g, Supplementary Fig. 1). In line with our previous observations, L-Dopa treatment did not induce any significant changes in the proportion of excitatory synapses in neurons lacking TTL or SVBP (71 ± 2% of spines vs 69 ± 2% of spines in TTL KO neurons and 66 ± 2% of spines vs 65 ± 2% of spines in SVBP KO neurons in control and L-Dopa treated cells, respectively; Fig. 3h).

These results demonstrate that L-Dopa incorporation into tubulin is responsible for the synaptic alterations observed in wild type neurons. The absence of these defects in TTL KO and SVBP KO neurons, where L-Dopa cannot be incorporated into microtubules, strongly suggests that the effect on synaptotoxicity is driven by abnormal L-Dopa microtubules.

### L-Dopa incorporation into tubulin modifies microtubule dynamics and their ability to invade dendritic spines

**Fig. 4:**
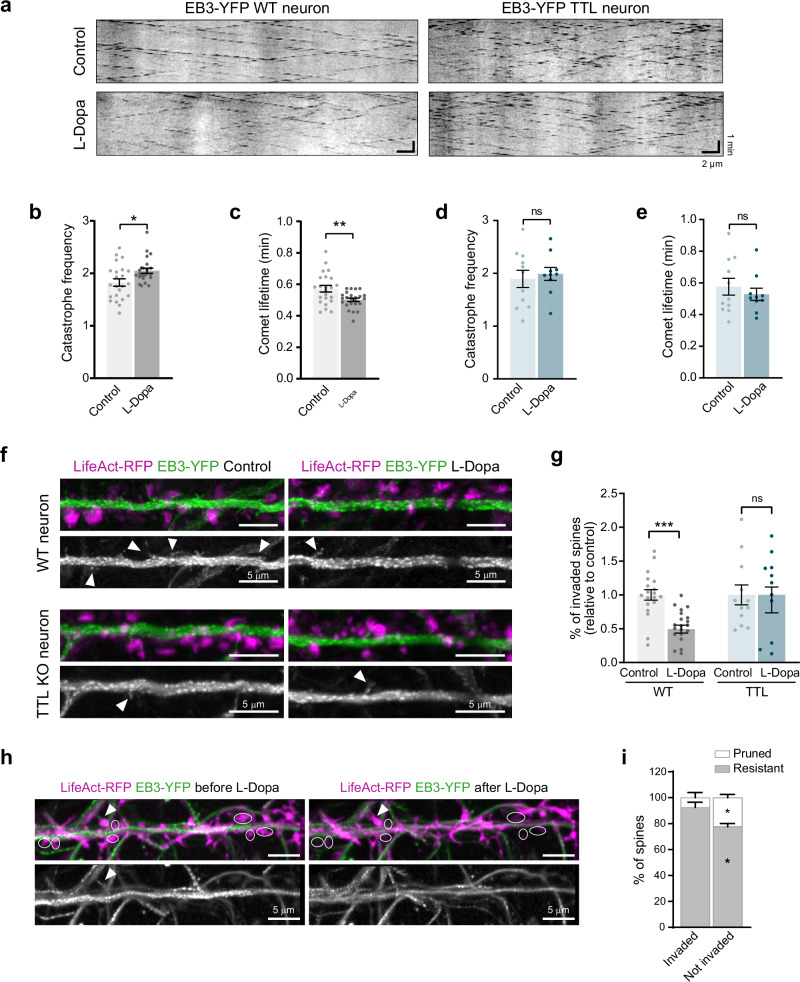
L-Dopa incorporation into tubulin modifies microtubule dynamics and their ability to invade dendritic spines. **a** Representative kymographs of EB3-YFP comets at the plus ends of growing microtubules in a dendritic segment of 18 DIV wild type (left) and TTL KO (right) hippocampal neurons, either untreated (Control, top) or treated with 0.4 mM L-Dopa (L-Dopa, bottom). Scale bar: horizontal, 2 µm; vertical, 1 min. **b**–**e** Quantification of microtubule dynamics parameters, including catastrophe frequency and comet lifetime for wild type (**b**, **c**) and TTL KO (**d**, **e**) neurons, respectively. Data represent mean ± SEM; *n* = 23, *n* = 24 control and L-Dopa-treated wild type neurons from three independent experiments and *n* = 11, *n* = 10 control and L-Dopa-treated TTL KO neurons from two independent experiments. Mann–Whitney test, *p < 0.05; **p < 0.01. **f** Representative maximal intensity projection of time-lapse confocal images showing dendritic segments of DIV 18 wild type (top) and TTL KO (bottom) hippocampal neurons expressing both LifeAct-RFP (magenta) and EB3-YFP (green at top; gray at bottom) in control and L-Dopa-treated neurons. White arrows indicate microtubules invading dendritic spines. Scale bar: 5 µm. **g** Percentage of spines invaded by microtubules in neurons treated with the vehicle (control) or L-Dopa (0.4 mM, 1 h). Values were normalized to the mean of the control cells. Data represent mean ± SEM; *n* = 19, *n* = 20 control and L-Dopa-treated wildtype and *n* = 11, *n* = 10 control and L-Dopa-treated TTL KO neurons, respectively, from at least three different experiments. Mann–Whitney test, ***p < 0.001. **h** Representative images showing dendritic segments of a neuron (DIV18) expressing LifeAct-RFP (magenta) and EB3-YFP (green at top; gray at bottom) before and after L-Dopa treatment. Scale bar: 5 µm. The white arrows point out the spines that were invaded by microtubules before L-Dopa treatment, and the circular ROIs indicate the dendritic spines that prune after L-Dopa treatment. **i** Percentage of pruned (white) or resistant (gray) spines. Graph represents the mean percentage of microtubule-invaded and non-invaded spines for either fate; *n* = 8 cells from two independent experiments. Two-way ANOVA, *p < 0.05.

Based on our findings that changes in dendritic spines and excitatory synapses relied on L-Dopa incorporation into tubulin, we subsequently investigated microtubule dynamics in the dendritic shaft of wild type and TTL KO hippocampal neurons, which lack the enzyme required for L-Dopa incorporation. We transiently expressed the microtubule plus-end binding protein EB3-YFP to track the dynamic behavior of microtubule plus ends (representative kymographs, Fig. 4a). We found that in wild type L-Dopa treated neurons, microtubule catastrophe frequency significantly increased with a corresponding decrease in comet lifetime compared to controls (1.83 ± 0.07 vs 2.05 ± 0.05, and 0.57 ± 0.02 min vs 0.50 ± 0.01 min for control and L-Dopa treated neurons, respectively; Fig. 4b, c), while EB3-comet growth length and speed were unaffected (4.4 ± 0.3 µm vs 4.0 ± 0.2 µm, and 9.2 ± 0.7 µm/min vs 9.1 ± 0.5 µm/min, for control and L-Dopa treated neurons, respectively; Supplementary Fig. 5a, b). None of these parameters were significantly modified after L-Dopa treatment in TTL KO neurons (1.9 ± 0.2 vs 2.0 ± 0.1, and 0.58 ± 0.05 min vs 0.53 ± 0.04 min for control and L-Dopa-treated neurons, respectively; Fig. 4d, e; Supplementary Fig. 5c, d). These observations demonstrate that the presence of L-Dopa on neuronal microtubules is responsible for alterations in their dynamics in dendrites.

In previous works, we demonstrated that abnormal synaptic microtubule dynamics, caused by a disrupted tubulin detyrosination/tyrosination cycle, reduced the number of microtubules entering into dendritic spines, leading to a significant synapse loss^16^. Here, we hypothesized that the presence of L-Dopa-enriched microtubules with altered dynamics might similarly affect spine invasion, potentially explaining the reduction in dendritic spines observed after L-Dopa treatment in wild type neurons. We analyzed microtubule invasions into individual spines of wild type and TTL KO cultured hippocampal neurons treated or not with L-Dopa. We imaged neurons infected with a custom-made construction of LifeAct-RFP-IRES-EB3-YFP to simultaneously label actin enriched in dendritic spines and track microtubule plus-end (Fig. 4f; Supplementary Video 1–4). L-Dopa treatment drastically reduced the percentage of spines invaded by microtubules in wild type neurons (1.00 ± 0.08% vs 0.54 ± 0.05% of spines invaded in 5 min in control and L-Dopa-treated neurons, respectively; Fig. 4f, g; Supplementary Video 1, 2), indicating that abnormal dynamic behavior of L-Dopa-microtubules has a major impact on synaptic microtubule invasion. As expected, L-Dopa treatment did not modify the percentage of microtubule-invaded spines in TTL KO neurons (1.0 ± 0.1% vs 1.0 ± 0.2% of spines invaded in control and L-Dopa-treated neurons, respectively; Fig. 4f, g; Supplementary Video 3, 4). To evaluate whether microtubule invasion is essential for preserving dendritic spine integrity in wild type neurons during L-Dopa treatment, we tracked and quantified the fate of individual spines, whether invaded or not by microtubules, before and after L-Dopa treatment (Fig. 4h, i; Supplementary Video 5, 6). Spines that were not invaded by microtubules were more likely to be pruned, while most microtubule-invaded spines remained unaffected (77 ± 2% vs 92 ± 4% resistant spines among non-invaded and invaded spines, respectively; Fig. 4i).

In summary, our results indicate that abnormal L-Dopa-microtubule dynamics disrupts microtubule entry into dendritic spines, thereby making them more susceptible to pruning. The consequent reduction in spine density could compromise the ability of the remaining dendritic spines to establish and maintain mature synapses.

## Discussion

In recent decades, growing evidence suggests that the neuronal cytoskeleton plays a critical role in maintaining synaptic function and structure. Microtubule invasion into dendritic spines has been involved in morphological changes in these structures, activity-dependent synaptic potentiation, and synaptic transmission^11,12,50^. Furthermore, maintaining a delicate balance between detyrosinated and tyrosinated tubulin is crucial for supporting both the functional and structural plasticity of synapses in the adult brain^16,31,40^.

This study describes for the first time the synaptic consequences of L-Dopa—the standard treatment for Parkinson’s disease—being incorporated into microtubules. Our findings demonstrate that, in wild type cultured mouse hippocampal neurons, L-Dopa treatment disrupts normal microtubule dynamics in the dendritic shaft and impairs microtubule entry into dendritic spines, resulting in a reduction of mature forms of dendritic spines and excitatory synapses.

We have previously described, using a brain-derived catecholaminergic cell line, that L-Dopa incorporation resulted in disrupted microtubule dynamic behavior^46^. While the dynamic behavior of microtubules—including growth and catastrophe events—are influenced by the cellular context and may vary across different neuronal populations, in this study we analyzed specifically the effects of L-Dopa incorporation into microtubules in the dendritic shaft of hippocampal neurons. It is well established that altering microtubule dynamics significantly affects dendritic spine density, likely due to an impaired ability of microtubules to invade these small protrusions^12,51,52^. Additionally, we recently reported that non-invaded spines are more susceptible to pruning in the presence of a synaptotoxic agent such as Aβ-oligomers, and that this effect could be reversed by restoring dynamic tyrosinated microtubule levels^16^. In this work, we observed an increase in the catastrophe frequency and a reduced lifetime of growing microtubules in the dendritic shaft of wild type neurons and, in line with the previous findings, we demonstrated that L-Dopa-microtubules failing to enter dendritic spines increased spine vulnerability. Therefore, following L-Dopa treatment, spines that were not invaded by microtubules were more likely to be pruned, resulting in a significant reduction in dendritic spine density. Furthermore, the incorporation of L-Dopa into microtubules not only reduced the number of mushroom spines (considered to be the most active and mature form of spines) but also decreased the number of excitatory synapses in the remaining spines, suggesting a global alteration in the synaptic compartment.

To confirm that the observed defects are a direct result of the incorporation of L-Dopa into microtubules, we investigated neurons lacking the key enzymes involved in the tubulin detyrosination/tyrosination cycle. We evaluated L-Dopa effects by comparing neurons of the same genotype, treated or not with L-Dopa. We did not observe any alterations in tubulin tyrosination/detyrosination levels in TTL KO neurons, where the missing TTL enzyme prevents L-Dopa incorporation into microtubules, nor in SVBP KO neurons, where the reduced detyrosinated tubulin leaves little substrate for L-Dopa incorporation. As expected, microtubule dynamics, dendritic spine invasion and excitatory synapse density remained unaffected after L-Dopa treatment in TTL KO neurons. Furthermore, the synaptic defects triggered by L-Dopa treatment were also absent in SVBP KO neurons, confirming that L-Dopa’s adverse effects on spine and synapse density in wild type neurons are indeed mediated through its incorporation into microtubules.

Recent studies have connected synaptic dysfunction to motor and nonmotor complications that arise in Parkinson’s disease patients after prolonged L-Dopa treatment. These complications include L-Dopa-induced dyskinesia and psychiatric complications, such as impulse control disorders, heightened anxiety, changes in social behavior and depression^3,53,54^. Interestingly, both the duration of the treatment and the dose of L-Dopa correlate with an increase in the risk of developing these complications^8,55^. We have previously described, using protein cellular extracts—containing endogenous carboxypeptidases at physiological concentrations—and purified tubulin in an in vitro system, that the incorporation of L-Dopa into tubulin was irreversible^47^. Now we have confirmed, using a cell-free system and recombinant proteins, that L-Dopa is not efficiently released from microtubules. We showed that the principal carboxypeptidase responsible for tubulin detyrosination in the brain, the VASH1-SVBP complex, is less efficient at releasing L-Dopa than tyrosine. The alteration in the binding behavior of the enzyme, induced by the presence of L-Dopa on the microtubular lattice, is the most plausible explanation for the observed differences in activity. This allows us to hypothesize that, following years of administration, the defective activity of carboxypeptidases on L-Dopa microtubules may result in a gradual accumulation of these abnormally modified microtubules, potentially replacing dynamic tyrosinated microtubules in neuronal cells, leading to a progressive alteration of synaptic transmission, which could accelerate neurodegeneration.

It is still unknown whether the presence of L-Dopa-modified microtubules or the reduction in the proportion of dynamic tyrosinated microtubules is the pathological feature that triggers synaptotoxicity. It has been shown that alterations in microtubule dynamics and the disruption of the microtubule cytoskeleton may increase the vulnerability of dopaminergic neurons to the pathogenesis and progression of Parkinson’s disease^56–58^. More importantly, it has been shown that a balanced detyrosination/tyrosination tubulin cycle is necessary for the maintenance of synaptic plasticity^16^, thus it is possible that the presence of L-Dopa-tubulin would contribute to synaptic dysfunction and damage by perturbing this fragile equilibrium. Furthermore, L-Dopa incorporation into microtubules may also be involved in other side effects beyond the brain. For instance, abnormal detyrosination of microtubules in cardiac cells is closely associated with heart failure^59^ and L-Dopa treatment has been linked to cardiovascular issues^60^, suggesting that L-Dopa incorporation into microtubules could also be implicated in this phenomenon.

Another alternative that may be subject to consideration is that L-Dopa, once incorporated into microtubules, would act as a novel posttranslational modification and contribute to the modulation of microtubules’ properties and their interaction with different cellular effectors. For instance, in previous work, we showed that L-Dopa-microtubules disrupt the binding of the molecular motor KIF5B, altering mitochondrial trafficking and distribution in neurons^47^. Proper mitochondrial positioning near dendritic spines is essential for maintaining synaptic energy needs and calcium homeostasis, required for signaling and plasticity, key processes that are disrupted in Parkinson’s disease^61,62^. The presence of L-Dopa-modified microtubules impairs mitochondrial transport and localization, potentially exacerbating synaptic deficits and contributing to neurodegeneration. Extensive studies will be necessary to elucidate this question and to assess the impact of L-Dopa incorporation into microtubules on the synaptic compartment in vivo.

Here, we describe that the incorporation of L-Dopa into tubulin induces several changes that could compromise neuronal integrity and may be relevant in the development of the pathological side-effects. These alterations have the potential to disrupt synaptic plasticity, compromise the formation and stability of mature synapses, and ultimately impact the functionality of the entire neural network. This mechanism could contribute to the reported loss of synaptic connections with prolonged L-Dopa administration^63,64^, impacting not only the substantia nigra, typically affected in Parkinson’s disease, but also other brain regions. Such widespread alterations in synaptic transmission could accelerate neurodegeneration across the brain, exacerbating the progression of the disease and L-Dopa-induced complications.

Our study has limitations that open avenues for future research. The clinical relevance of our findings would be greatly strengthened by analyzing postmortem brain samples from Parkinson’s disease patients treated with L-Dopa. This would allow us to assess tubulin tyrosination status and the presence of L-Dopa-tubulin across different neuronal populations. Alternatively, the use of animal models of L-Dopa-induced dyskinesia could help determine whether L-Dopa-induced microtubule modifications similarly contribute to synaptic instability in vivo, and to confirm whether these effects are restricted only to the dopaminergic region or globally affect the entire brain. We acknowledge this gap and plan to address it in future investigations to further elucidate the role of tubulin detyrosination/tyrosination in L-Dopa-induced neurological effects.

Overall, this study highlights tubulin detyrosination/tyrosination as a potential mechanistic pathway underlying the regulation of synaptic stability and neuronal function. This insight offers a new perspective on some neurological deficits associated with prolonged L-Dopa therapy in Parkinson’s disease, introducing the detyrosination/tyrosination cycle enzymes and microtubule dynamics as potential therapeutic targets.

## Methods

### Animals

All experiments involving mice were conducted in accordance with the policy of the Grenoble Institut des Neurosciences and in compliance with the French legislation and European Union Directive of 22 September 2010 (2010/63/UE). TTL heterozygous (TTL+/-) mice and mice homozygous for an inactivated small vasohibin binding protein allele (referred to as SVBP-KO) were obtained as previously described^31,39^ and maintained in a C57BL/6 genetic background by recurrent back-crosses with C57BL6 animals from Charles River Laboratories.

### Genotyping

PCR amplifications were performed on alkaline lysates of toe clips or tail cuts of E18.5 mouse embryos. Briefly, mouse tissue was incubated for 30 min at 95 °C in alkaline solution (NaOH 25 mM, EDTA 0.2 mM, pH 12.0). Neutralization was performed by adding 40 mM Tris, pH 5.0. Lysates were then analyzed by PCR with corresponding primers and EconoTaq PLUS Green Mix (Euromedex). Primers pairs for testing the TTL mouse strain were 5 ′ -GGCGACTCCATGGAGTGGTGG-3 ′ and 5 ′CCCAACAT-CACATTCTCCAAATATCAAAG-3 ′ (TTL wild type, 1032 bp) and 5 ′GATTC-CCACTTTGTGGTTCTAAGTACTG 3′ and 5′CCCAACATCACATTCTCCAAATATCAAAG-3 ′ (TTL KO, 900 bp). The four primers were used in a single reaction. Primers pairs for testing SVBP mouse strain were 5 ′GATCCACCTGCCCGGAAA 3 ′ and 5 ′TTTCTTCCAGCACCCTCTCC 3 ′ (SVBP wild type, 170 bp) and 5 ′TTTCTTCCAGCACCCTCTCC 3 ′ and 5 ′CAAACCATGGATCCACGAAA 3 ′ (SVBP KO, 167 bp). These latter reactions were done separately^32^. The following amplification protocols were used: TTL, 95 °C for 5 min, 35 cycles of [95 °C for 1 min, 50 °C for 1 min, 72 °C for 1 min], 72 °C for 2 min; SVBP, 95 °C for 5 min, 33 cycles of [95 °C for 30 s, 50 °C for 30 s, 72 °C for 30 s], 72 °C for 2 min. DNA was analyzed on 1.2% and 2% agarose gels for TTL and SVBP, respectively.

### Plasmids

For lentiviral experiments, a vector eGFP-pWPT (Addgene #12255, a kind gift from D. Trono) was used to express eGFP and a homemade vector containing LifeAct-RFP - IRES - EB3-YFP was used to express both LifeAct-RFP and EB3-YFP. For the last one, the cDNAs encoding LifeAct (aa 1–17 of *Saccharomyces cerevisiae* ABP140, NP_014882) C-terminally fused to RFP, and encoding human EB3 (NP_001289979) C-terminally fused to YFP were cloned in pLV-mCherry vector. During the cloning process, mCherry cDNA was removed and replaced by an IRES (encephalomyocarditis virus internal ribosome entry site) sequence to allow EB3-YFP expression. PCR amplification and cloning of cDNAs were performed with Phusion DNA polymerase (Thermo Scientific) and In-Fusion HD Cloning kit (Clontech), respectively. The construct was verified by sequencing (Eurofins and Genewiz) and purified with HiPure Plasmid Maxiprep kits (Invitrogen).

### Lentivirus production

Lentiviral particles were produced using the second-generation packaging system. Lentivirus encoding eGFP-pWPT or LifeAct-RFP - IRES - EB3-YFP were produced by co-transfection with the psPAX2 and pCMV-VSV-G helper plasmids (Addgene plasmids # 12260 and # 8454, gifts from Didier Trono and Bob Weinberg, respectively), into HEK293T cells (ATCC-CRL-3216) using the calcium phosphate transfection method. Viral particles were collected 48 h after transfection by ultra-speed centrifugation, prior to aliquoting and storage at −80 °C.

### Primary hippocampal neuronal cultures

E18.5 mouse embryos, regardless of their genotype, were processed individually. Hippocampi were dissected and digested in 0.25% trypsin in Hanks’ balanced salt solution (HBSS, Invitrogen, France) at 37 °C for 15. min. After manual dissociation, cells were plated at a concentration of 5000–15,000 cells/cm^2^ on 1 mg/mL poly-L-lysine-coated coverslips for fixed samples, or on Ibidi 35 mm glass-bottom dishes for live imaging or plastic petri dishes for immunoblot analysis. Neurons were incubated 2 h in DMEM-10% horse serum and then changed to MACS neuro medium (Miltenyl Biotec) with B27 supplement (Invitrogen, France). One third of the medium was changed every 3–4 days up to 3 weeks in culture.

### Lentivirus infection

To perform dendritic spine quantification, a lentivirus containing eGFP at a multiplicity of infection of 5 was added to the cells at 7 DIV. Hippocampal neurons were incubated until 18 DIV at 37 °C, 5% CO_2_ in a humidified incubator and then fixed with 4% paraformaldehyde in 4% sucrose-containing PBS for 20 min. To analyze microtubule invasion into the spines, mouse hippocampal neurons were grown on 35 mm glass-bottom live imaging dishes (Ibidi) and infected at 7 DIV with a lentivirus containing LifeAct-RFP and EB3-YFP cDNA at a multiplicity of infection of 5.

### L-Dopa treatment

L-Dopa was incorporated into cultured hippocampal neurons as previously described^46,47^. Briefly, the culture medium was substituted with Hank’s Balanced Salt Solution (HBSS; composition: 137 mM NaCl, 5 mM KCl, 0.8 mM MgSO_4_, 0.33 mM Na_2_HPO_4_, 0.44 mM KH_2_PO_4_, 0.25 mM CaCl_2_, 1 mM MgCl_2_, 0.15 mM Tris-HCl, pH 7.4), supplemented with freshly prepared 1 mM sodium butyrate. Additionally, 1 μM tolcapone (Sigma-Aldrich) and 50 μM carbidopa (Sigma-Aldrich) were added to the solution. The solution was then either supplemented with or without 0.4 mM L-Dopa (Sigma-Aldrich). The cells were then incubated for 1 h at 37 °C in a humidified atmosphere with 5% CO_2_.

### Immunofluorescence and antibodies

For immunocytochemistry, cells were fixed with 4% paraformaldehyde in 4% sucrose-containing PBS for 20 min and permeabilized with 0.2% Triton X-100/ PBS for 5 min. Fixed cells were then incubated with primary antibodies for 3 h in 0.1% PBS/Tween and then with fluorophore-conjugated secondary antibodies for 1 h at room temperature. Primary antibodies were mouse monoclonal anti PSD-95 (clone K28/43, NeuroMab) diluted 1:500, rabbit polyclonal anti Synaptophysin (SYP (H-93), Santa Cruz Biotechnology) diluted 1:500. Secondary antibodies were coupled to Alexa-488, to Alexa-565 or to Alexa-647 (Jackson Immuno-Research Laboratories).

### Imaging of dendritic spines

For cultured samples, hippocampal neurons from wild type, SVBP KO and TTL KO embryos were infected with eGFP-containing lentivirus and fixed at DIV18 prior to mounting with DAKO antifade mounting reagent (Agilent S3023). Dendritic segments visualized by eGFP were obtained using an inverted microscope (Axio Observer, Zeiss) coupled to a spinning-disk confocal system (CSU-W1-T3, Yokogawa) and a LiveSR (super resolution) module connected to a wide-field electron-multiplying charge-coupled device (CCD) camera (ProEM+1024, Princeton Instrument). Serial optical sections with pixel dimensions of 0.104 × 0.104 μm were collected at 0.2 µm intervals for 10-15 stacks, using a ×63 oil-immersion objective (NA 1.46). Dendritic spine analysis (spine counting and shape classification) was performed on reconstructed neurons from 3D confocal images using Neuronstudio^16,65^. The linear density was calculated by dividing the total number of spines present on assayed dendritic segments by the total length of the segments. At least two dendritic regions of interest were analyzed per cell from at least three independent embryos per culture in each experimental condition.

### Imaging of excitatory synapses

For immunodetection of excitatory synapses in eGFP-expressing neurons (DIV 18), cells were incubated 1 h in HBSS with or without L-Dopa (4 mM, 1 h), as previously described. After treatment, HBSS was removed and conditioned MACs complete medium was added to allow the cells to recover for 1 h. Subsequently, cells were fixed and immunolabeled with PSD-95 and Synaptophysin antibodies. Fluorescent images were acquired with an inverted microscope (Axio Observer, Zeiss) coupled to a spinning-disk confocal system (CSU-W1-T3, Yokogawa) and a LiveSR module connected to a wide-field electron-multiplying charge-coupled device (CCD) camera (ProEM+1024, Princeton Instrument). Serial optical sections with pixel dimensions of 0.104 × 0.104 μm were acquired with 4 z-stack planes at 0.7 µm step size using a ×63 oil-immersion objective (NA 1.46) and Metamorph software. Images were enhanced for small structures with the LoG3D ImageJ plugin using a 2-pixel radius, and thresholded to create a mask for: eGFP (cell filler and contour marker), Synaptophysin (pre-synaptic compartment) and PSD-95 (post-synaptic compartment). The mask corresponding to the overlap of the three markers (colocalization mask) was superposed on the eGFP image. The synaptic puncta present in dendritic spines were manually counted. Mask creation and counting were done blind to the genotype and treatment.

### Live imaging of microtubule dynamics and dendritic spine invasion

LifeAct-RFP and EB3-YFP expressing hippocampal neurons (DIV 18) were incubated 1 h in HBSS with or without L-Dopa (4 mM, 1 h), as previously described. Initial conditioned MACs complete medium was added before transferring the cells to an inverted microscope (Axio Observer, Zeiss) coupled to a spinning-disk confocal system (CSU-W1-T3, Yokogawa) connected to a wide-field electron-multiplying charge-coupled device (CCD) camera (ProEM+1024, Princeton Instrument) with a ×63/1.46 oil objective and maintained at 37 °C and 5% CO_2_. Movies of microtubule dynamics were acquired at 5 s/frame for 5 min with 4 z-stack planes at 0.7 µm step size by Metamorph software.

Maximum projections of movies were performed and analyzed in ImageJ. The percentage of the spines invaded was calculated as the number of spines invaded by microtubules during a 5 min film/total number of spines in the imaging field^16^. To analyze microtubule dynamics, Kymographs were generated by drawing a region of interest at the middle part of the dendrite. Parameters describing the dynamics of microtubules within the selected region were measured using a homemade plugin and defined as follows: catastrophe frequency: number of full tracks/total duration of growth; growth length: comet movement length in μm; comet lifetime: duration of growth; growth rate: growth length/comet lifetime^16^.

### Analysis of spine structural plasticity

Dendritic spines were examined in the same imaging field before (0 h) and after (1 h) L-Dopa treatment. Spines were manually counted as either invaded or not invaded by EB3 comets, using ImageJ Software. Next, the percentages of spines that persisted or disappeared following L-Dopa treatment were calculated. These percentages were based on the total number of spines initially classified as invaded or not invaded by EB3 comets in the same field before the addition of L-Dopa.

### Biochemical analysis of the microtubule network of cultured neurons

Hippocampal neurons (17 DIV) treated with or without L-Dopa (0.4 mM), as previously described, were lysed in Laemmli buffer. L-Dopa incorporation into microtubules was calculated by semi-quantitative immunoblot analysis of tyrosinated, detyrosinated and Δ2 tubulin levels. Briefly, neurons and HEK293T cells expressing each modified mCherry-α-tubulin were analyzed in the same immunoblot. To determine the sensitivity of each modification-specific antibody, we calculated the ratio between the modification-specific antibody signal and the total α-tubulin signal obtained with the corresponding modified mCherry-α-tubulin^30^. Then signals obtained with neuron extracts were normalized to total α-tubulin levels and to antibody sensitivity in the experiment. Antibodies used: rat monoclonal anti Tyr-tubulin (YL1/2) diluted 1:10,000, rabbit polyclonal anti-deTyr tubulin diluted 1:10,000, rabbit polyclonal anti-Δ2 tubulin diluted 1:10,000, rabbit polyclonal homemade antibody anti-Δ3 tubulin diluted 1:8000, and mouse monoclonal antibody against α-tubulin (clone α3A1) and against β-tubulin diluted 1:10,000. All anti-tubulin antibodies were described in Aillaud et al.^30^. Secondary antibodies were coupled to Alexa-488, to Alexa-565 or to Alexa-647 (Jackson Immuno-Research Laboratories). Several neuronal cultures were used as indicated in the figure legends, and for each sample, at least 2 independent blots were performed.

### Constructs and enzymes purification for TIRF and immunofluorescence studies

We used the human VASH1 (NP_055724)/human SVBP (NP_955374) complex fused to the sfGFP tag. The VASH1 constructs used in this study were: VASH1 full-length (VASH1, residues 1–365) and the catalytically inactive VASH1 full length (deadVASH1, residues 1–365, C169A)^49^. Protein expression and purification of the various His-tagged VASH1–SVBP complexes were performed as previously described^49^.

### Preparation of Tyr- and L-Dopa-enriched tubulin and Taxol-stabilized microtubules

Preparation of brain tubulin and its labeling with either biotin or ATTO-565 fluorophore (ATTO-TEC Gmbh) was performed according to Ramirez-Rios et al.^49^.

Aliquots of 2.5 mg of purified brain tubulin were incubated for 15 min at 30 °C in the presence of 2 U⋅mL-1 carboxypeptidase A (CPA) to remove the endogenous C-terminal tyrosine and increase the levels of detyrosinated tubulin that will act as substrate for tyrosine or L-Dopa incorporation^66^. The CPA enzyme was subsequently inhibited by the addition of 20 mM dithiothreitol (DTT), and the tubulin samples were passed through a PD-10 desalting column filled with Sephadex G-25 resin. The eluted tubulin was centrifuged at 4 °C using a Vivaspin 500 concentrator (Sartorius) to achieve a final concentration of 10 mg⋅mL^-1^.

Parallel incorporation of L-Dopa or tyrosine into the purified detyrosinated tubulin was carried out using an incubation system composed of: 10 mg⋅mL^-1^ tubulin, 1 mM DTT, 2.5 mM ATP, 12.5 mM MgCl_2_, 100 mM KCl, 12 mM ascorbic acid, and 0.5 mM L-Dopa (Sigma-Aldrich) or 1 mM L-Tyrosine (Sigma-Aldrich), respectively^67^, in BRB80 buffer (80 mM Pipes-KOH at pH 6.8, 1 mM MgCl_2_, 1 mM EGTA). 0.25 mg⋅mL^-1^ of the recombinant TTL enzyme was added to the system and incubated for 30 min at 35 °C. After the incorporation of L-Dopa or tyrosine, the samples were cooled to 4 °C for 10 min and centrifuged at 200,000×*g* for 10 min. The supernatants were collected and incubated for 1 h at 35 °C in the presence of 30% glycerol, 1 mM GTP, and 5 mM MgCl_2_ to induce microtubule polymerization.

Microtubules enriched in Tyr- or L-Dopa-tubulin were centrifuged through a 60% glycerol cushion (v/v in BRB80) at 200,000×*g* for 30 min at 35 °C. The pellet was resuspended in 15 μL of BRB80 buffer, and all tubulin proteins were stored in liquid nitrogen.

Taxol-stabilized microtubules were prepared, as described previously^49^, by polymerizing 45 µM tubulin (composed of 65% of Tyr- or L-Dopa-tubulin, 30% biotinylated brain tubulin, and 5% ATTO-565–labeled brain tubulin) in BRB80 supplemented with 1 mM GTP. To allow the formation of microtubules, the mixture was incubated for 1 h at 35 °C. Subsequently, 100 μM Taxol was added and incubation was continued for 30 min. Tyr- or L-Dopa-microtubules were then centrifuged 10 min at 200,000×*g* and resuspended in BRB80 supplemented with 10 μM Taxol.

### TIRF in vitro assay

Flow chambers for TIRF imaging assays were prepared as previously described^49^. Subsequently, a solution of microtubules enriched in either Tyr- or L-Dopa-tubulin was perfused into and incubated at room temperature for 5–7 min. Unbounded microtubules were removed by three washes of 100 µL of 1% BSA/10 µM Taxol in BRB80, supplemented with 10 μM Paclitaxel. Finally, 30 µL of a solution containing 25 pM of the catalytically inactive sfGFP–VASH1–SVBP complex^49^ in BRB80 (supplemented with 82 µg/mL catalase, 580 µg/mL glucose oxidase, 1 mg/mL glucose, 4 mM DTT, 0.5 mM Taxol, 0.01% methylcellulose 1500 cp) was perfused. The chamber was sealed, and images were recorded within the first 30 min following addition of the assay-mix solution on an inverted microscope (Eclipse Ti, Nikon). The microscope was equipped with a Perfect Focused System, a CFI Apochromat TIRF ×100/1.49 N.A. oil immersion objective (Nikon), a warm stage controller (Linkam Scientific) and a Technicoplast chamber to maintain the temperature, an objective heater (OkoLab), an iLas2 TIRF system (Roper Scientific), and a sCMOS camera (Prime95B, Photometrics) controlled by MetaMorph software (version 7.10.3, Molecular Devices). For dual-view imaging, an OptoSplit II bypass system (Cairn Research) was used as an image splitter and illumination was provided by 488- and 561-nm lasers (150 and 50 mW, respectively). Temperature was maintained at 35 °C for all imaging purposes. Acquisition rate was one frame each 50 ms exposure (in streaming acquisition) during 45 s.

### In vitro assay of detyrosination activity using immunofluorescence

VASH1-SVBP activity was measured as previously described^49^. Immunofluorescence in vitro assays were made using the same perfusion chambers and TIRF experiments conditions as above. After the addition of the assay-mix solution, incubation was done at 37 °C for 30 min followed by three washes with 10 µM Taxol and 1% BSA in BRB80 (wash buffer). The incubation with primary antibodies (rat anti-tyrosinated tubulin (YL1/2 = anti-Tyr, 1:6000) and rabbit anti-detyrosinated tubulin (anti-deTyr, 1:1000)) was done for 15 min, followed by three washes with 100 µL of wash buffer. Subsequently, secondary antibodies (anti-rat coupled to Alexa Fluor 488 (Jackson ImmunoResearch, 712-545-153) and anti-rabbit coupled to Cyanine 3 (711-165-152; Jackson ImmunoResearch), both diluted to 1:500) were added and incubated for 15 min, followed by three washes with 100 µL of wash buffer. Images were obtained using a LEICA DMI600/ROPER microscope controlled by Metamorph Video software using the same illumination conditions. For each condition, at least three independent experiments with different protein preparations were done.

### Data analysis

Immunofluorescence and binding tracking of single VASH1–SVBP molecules for estimation of activity and binding parameters were measured using FIJI software and a homemade plugin KymoTool^68^.

### Statistical analysis

All data are presented as mean ± SEM. The number of replicates and tests used is indicated in the figure legends for each analysis. Statistical significance of differences between conditions was calculated with Prism 8.0 (GraphPad Software). Comparisons were made using at least two or three independent experiments, each of which includes multiple neurons from individually processed embryos. Mean differences were considered significant at *p < 0.05; **p < 0.01; ***p < 0.001 and ****p < 0.0001.

## Supplementary information

Supplementary Information

Supplementary Video1

Supplementary Video2

Supplementary Video3

Supplementary Video4

Supplementary Video5

Supplementary Video6

## Data Availability

No datasets were generated or analyzed during the current study.
