# Supplementary material for: L-Dopa-modified microtubules lead to synapse instability in cultured neurons: possible implications in Parkinson’s disease therapy
Source: NPJ Parkinsons Dis. 2025 Oct 17;11:298. doi: 10.1038/s41531-025-01143-4 (PMC12534460; doi:10.1038/s41531-025-01143-4)

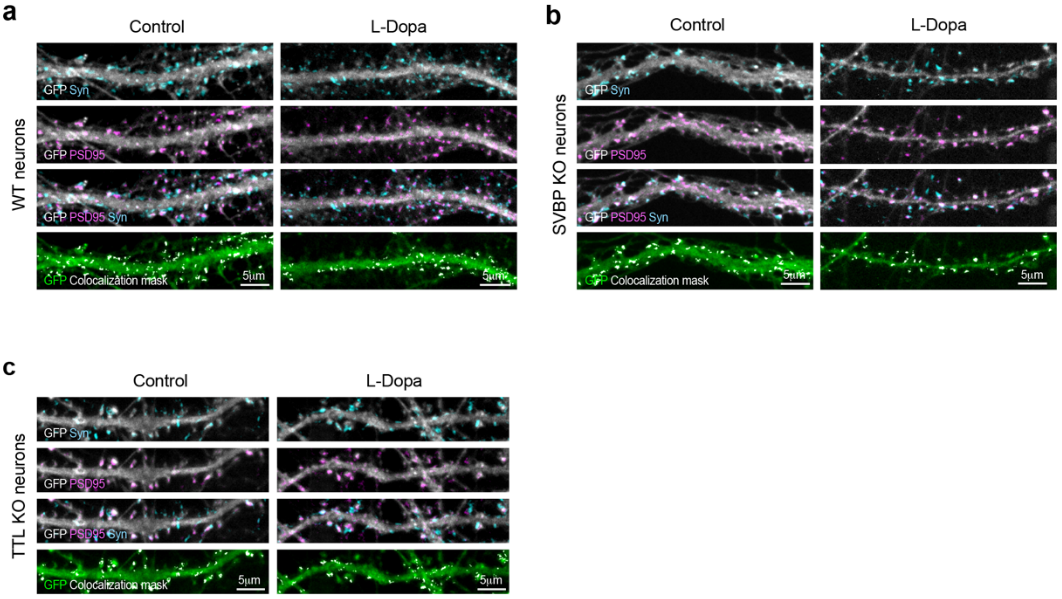

Supplementary Figure 1 L-Dopa incorporation into microtubules reduces excitatory synapses in cultured hippocampal neurons. Confocal images of magnified dendritic segments from control and L-Dopa treated **(a)** WT, **(b)** SVBP KO, and **(c)** TTL KO hippocampal neurons expressing soluble GFP (gray/green), and stained with anti-synaptophysin (cyan) and-anti-PSD95 (magenta) antibodies. The last image of each panel corresponds to the dendritic segment (GFP, green) superposed with a mask (white) indicating the pixels that simultaneously represent PSD-95, synaptophysin and GFP labels. Scale bar: 5 µm. **(a)** Related to Fig. 1; **(b - c)** related to Fig. 3.

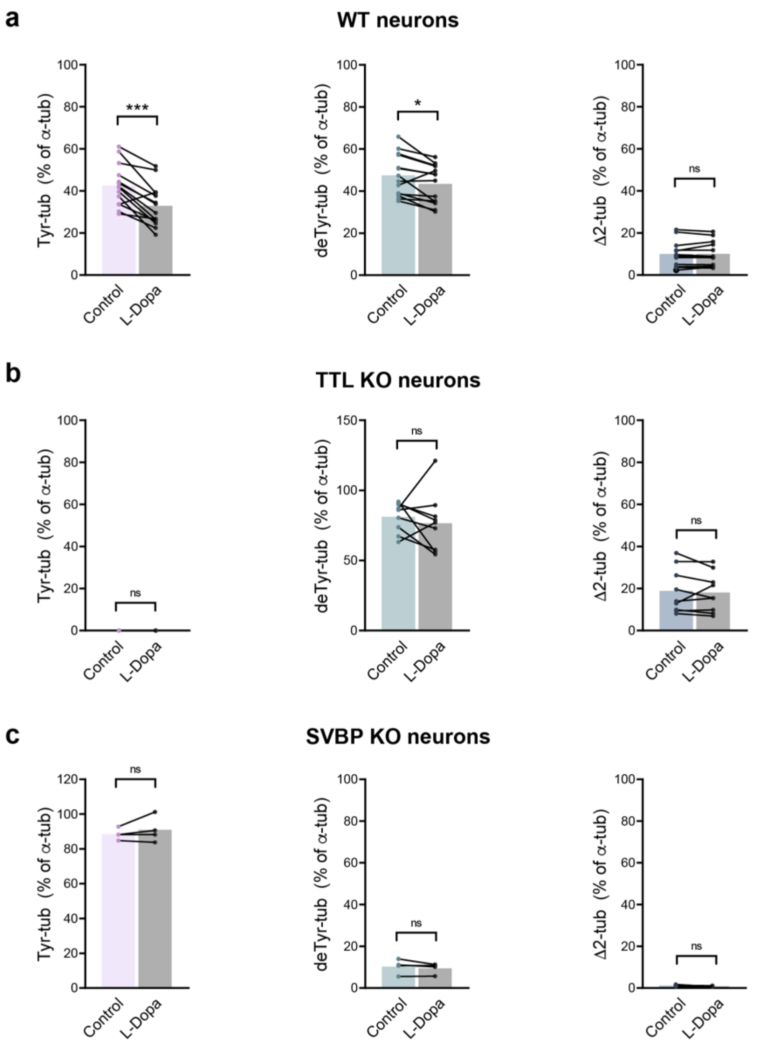

Supplementary Figure 2 L-Dopa treatment modifies the tyrosination state of α-tubulin only in wild type neurons. (a - c) Variation in the tyrosinated (Tyr-tub), detyrosinated (deTyr-tub) and Δ2 (Δ2-tub) α-tubulin levels in wild type **(a)**, TTL KO **(b)** and SVBP KO **(c)** neurons (DIV 17) treated with L-Dopa (0.4 mM) or the vehicle (Control). The content of the different α-tubulin forms was estimated after normalization to total α-tubulin levels and antibody sensitivity, as described in the methods section. Graphs represent the mean values, each pair corresponds to neurons from the same embryo treated with or without L-Dopa; neurons from n = 14 WT, n = 9 TTL KO and n = 4 SVBP KO embryos from at least three independent experiments. Paired t-test; *** $p < 0.001$; ** $p < 0.01$.

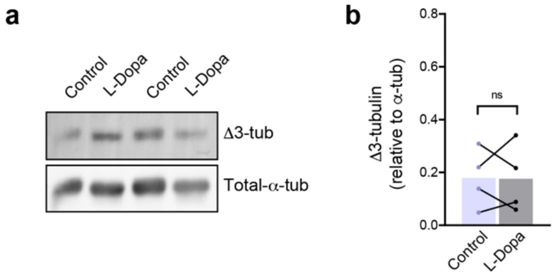

Supplementary Figure 3 L-Dopa treatment does not modify Δ3-tubulin levels. (a) Representative immunoblot of protein extract from WT hippocampal neurons (17 DIV) treated with L-Dopa (0.4 mM, 1h) or the vehicle (control), showing Δ3- (Δ3-tub) and total- (Total-α-tub) α-tubulin levels. **(b)** Quantitative analysis. The content of Δ3-tubulin was estimated after normalization to total α-tubulin levels in the same sample. Paired t-test; n = 4 neuron samples from two independent experiments.

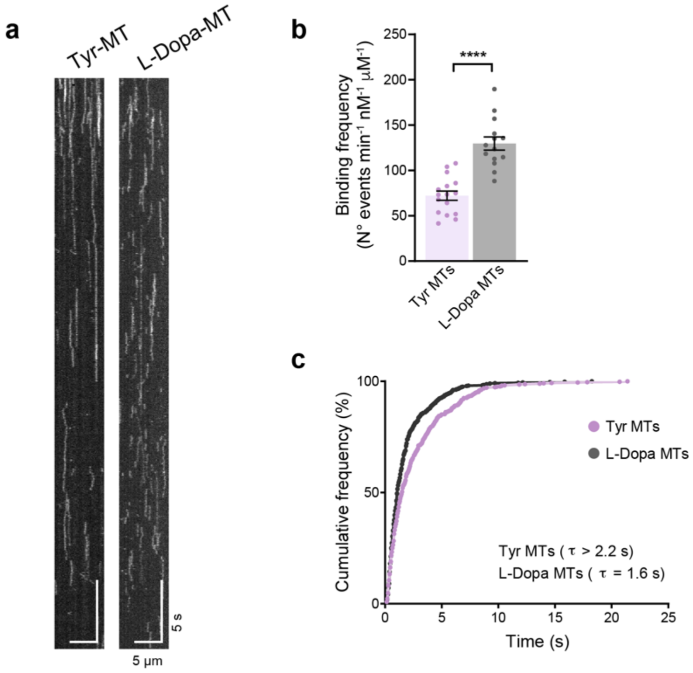

Supplementary Figure 4 Altered binding behavior of VASH1-SVBP on L-Dopa-enriched microtubules. (a) Representative kymographs of single molecules of catalytically inactive sfGFP-tagged VASH1–SVBP (deadVASH1-SVBP, 50 pM) bound to Taxol-stabilized microtubules enriched in tyrosinated (Tyr MTs) or L-Dopa (L-Dopa MTs) tubulin. Scale bars: horizontal, 5 µm; vertical, 5 s. **(b)** Number of binding events of the deadVASH1-SVBP to tyrosinated (Tyr MTs) or L-Dopa (L-Dopa MTs) microtubules. **(c)** Cumulative frequency of the residence times measured in TIRF movies taken during the 30 min following addition of enzyme complexes to tyrosinated or L-Dopa microtubules. The mean residence time (τ) is obtained by fitting the curve with a mono-exponential function[49]. Each point corresponds to an individual microtubule. Data represent mean ± SEM; n = 15 microtubules per condition. Student's t-test; ** $p < 0.01$.

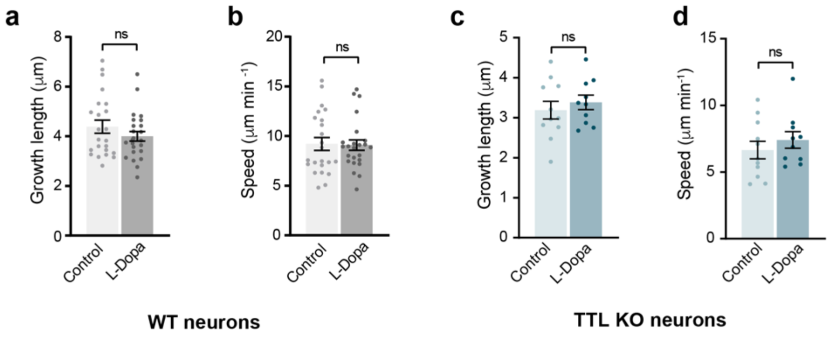

Supplementary Figure 5 L-Dopa treatment does not modify EB3-comet growth length or speed in wild type nor TTL KO hippocampal neurons. Quantification of microtubule dynamics parameters including comet growth length **(a, c)** and speed **(b, d)** in dendritic segments of wild type and TTL KO hippocampal neurons, respectively. Data represent mean ± SEM; n = 23, n = 24 control and L-Dopa-treated wild type neurons, and n = 11, n = 10 control and L-Dopa-treated TTL KO neurons, from four and two independent experiments, respectively. Mann-Whitney test, ns = not significant.

Supplementary Video 1 Microtubule dynamics in WT Control hippocampal neurons. Time-lapse video microscopy of the dendritic segment of a wild-type hippocampal neuron (DIV 18) expressing LifeAct-RFP (magenta in the upper panel) and EB3-YFP (green in the upper panel, gray in the lower panel), treated with the vehicle. Images were collected at 1 frame every 5 s for 5 min. Arrows indicate microtubules invading dendritic spines. Scale bar = 5 μm. Related to Figure 4.

Supplementary Video 2 Microtubule dynamics in WT L-Dopa treated hippocampal neurons. Time-lapse video microscopy of the dendritic segment of a wild-type hippocampal neuron (DIV 18) expressing LifeAct-RFP (magenta in the upper panel) and EB3-YFP (green in the upper panel, gray in the lower panel), treated with L-Dopa. Images were collected at 1 frame every 5 s for 5 min. Arrows indicate microtubules invading dendritic spines. Scale bar = 5 μm. Related to Figure 4.

Supplementary Video 3 Microtubule dynamics in TTL KO Control hippocampal neurons. Time-lapse video microscopy of the dendritic segment of a TTL KO hippocampal neuron (DIV 18) expressing LifeAct-RFP (magenta in the upper panel) and EB3-YFP (green in the upper panel, gray in the lower panel), treated with the vehicle. Images were collected at 1 frame every 5 s for 5 min. Arrows indicate microtubules invading dendritic spines. Scale bar = 5 μm. Related to Figure 4.

Supplementary Video 4 Microtubule dynamics and their ability to invade dendritic spines in TTL L-Dopa treated hippocampal neurons. Time-lapse video microscopy of the dendritic segment of a TTL KO hippocampal neuron (DIV 18) expressing LifeAct-RFP (magenta in the upper panel) and EB3-YFP (green in the upper panel, gray in the lower panel), treated with L-Dopa. Images were collected at 1 frame every 5 s for 5 min. Arrows indicate microtubules invading dendritic spines. Scale bar = 5 μm. Related to Figure 4.

Supplementary Video 5 Microtubule dynamics in WT hippocampal neurons before L-Dopa treatment. Time-lapse video microscopy of the dendritic segment of a wild-type hippocampal neuron (DIV 18) expressing LifeAct-RFP (magenta in the upper panel) and EB3-YFP (green in the upper panel, gray in the lower panel), before L-Dopa

treatment. Images were collected at 1 frame every 5 s for 5 min. Arrows indicate microtubules invading dendritic spines. Scale bar = 5 μm. Related to Figure 4.

Supplementary Video 6 Microtubule dynamics in WT hippocampal neurons after L-Dopa treatment. Time-lapse video microscopy of the dendritic segment of a wild-type hippocampal neuron (DIV 18) expressing LifeAct-RFP (magenta in the upper panel) and EB3-YFP (green in the upper panel, gray in the lower panel), after L-Dopa treatment. Images were collected at 1 frame every 5 s for 5 min. Arrows indicate microtubules invading dendritic spines. Scale bar = 5 μm. Related to Figure 4.

Source data Figure 1 and Supplementary Figure 3

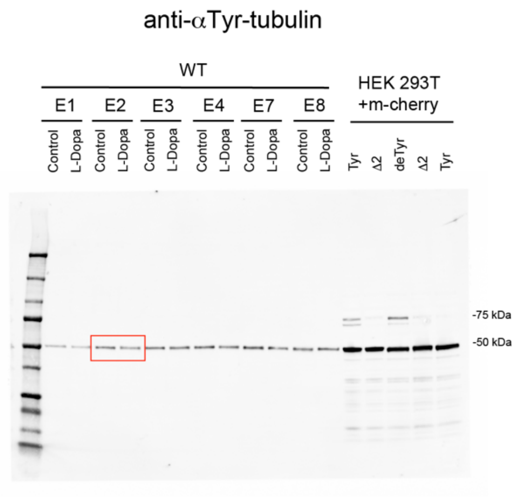

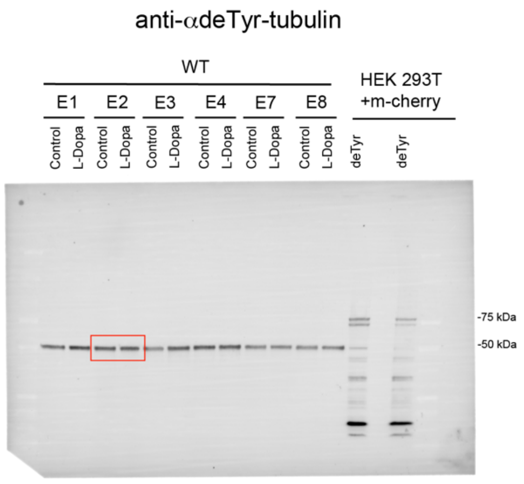

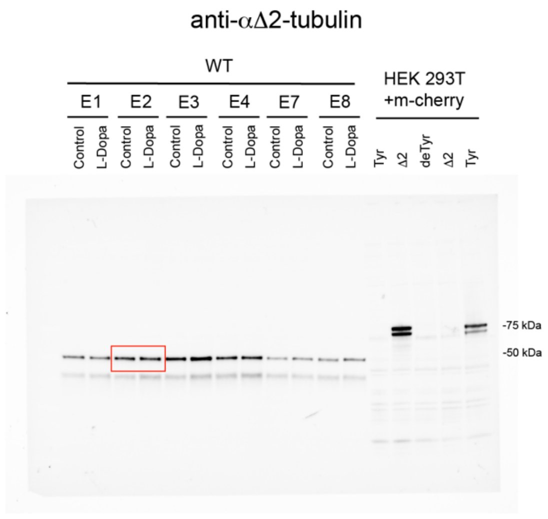

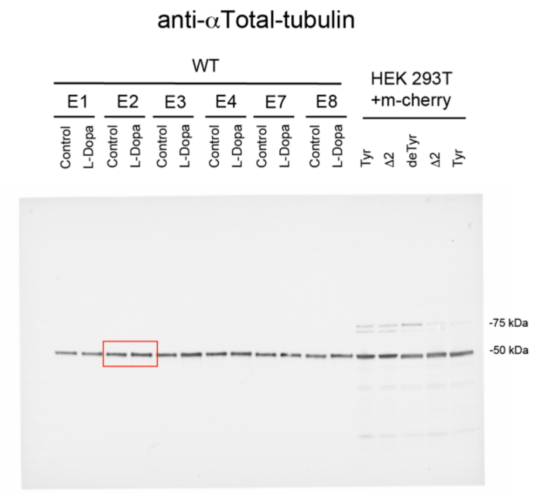

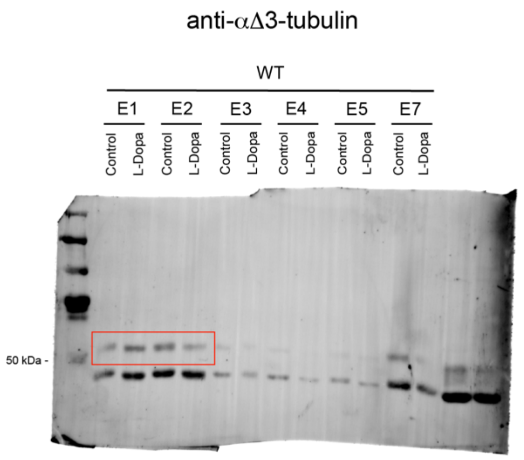

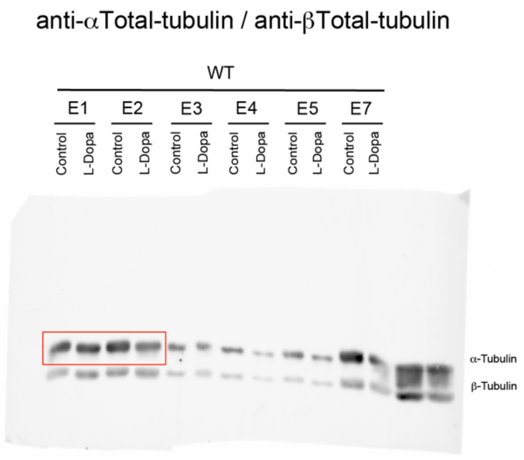

Source data Figure 2

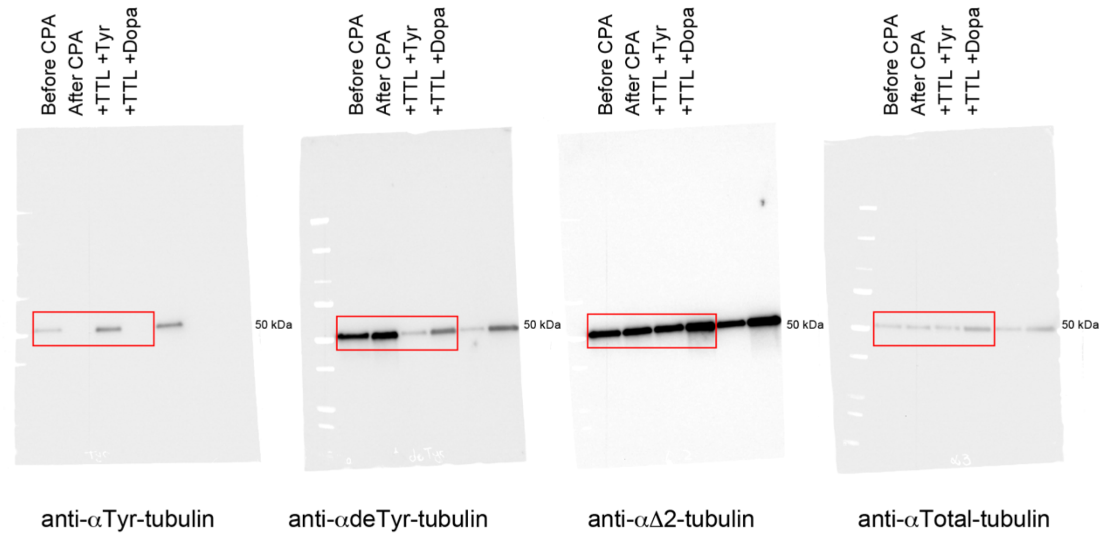

Source data Figure 3

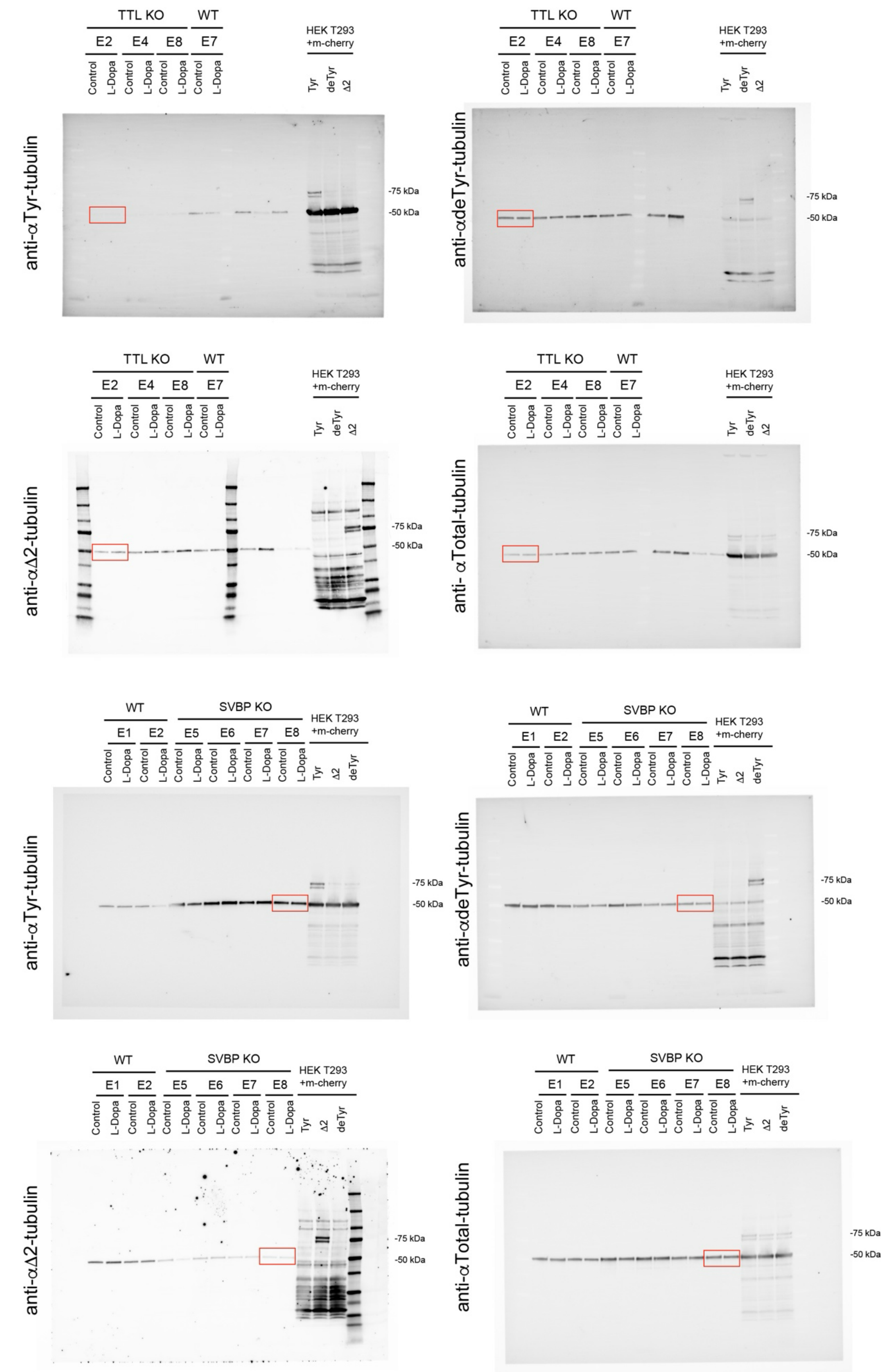

Supplement: Supplementary file 1 — Supplementary Information [file 41531_2025_1143_MOESM1_ESM.pdf]
